# Prevalence of antimicrobial‐resistant *Escherichia coli* in endangered Okinawa rail (*Gallirallus okinawae*) inhabiting areas around a livestock farm

**DOI:** 10.1002/vms3.194

**Published:** 2019-08-26

**Authors:** Sawako Ishibashi, Daisuke Sumiyama, Tomoko Kanazawa, Koichi Murata

**Affiliations:** ^1^ College of Bioresource Sciences Nihon University Fujisawa Kanagawa Japan

**Keywords:** antimicrobial‐resistant, endangered species, *Escherichia coli*, *Gallirallus okinawae*, Okinawa rail

## Abstract

Antimicrobial resistance (AMR) is an important issue for public, animal and environmental health. It has been suggested that livestock farms could be a source origin of AMR, and some wild animals that inhabit this area may play an important role in the spread of AMR in the natural environment. The prevalence of AMR in *Escherichia coli* was examined from Okinawa rails (*Gallirallus okinawae*), an endemic bird in Okinawa Main Island, Japan. Forty‐eight faecal samples of wild Okinawa rails were collected from around a livestock farm area (LA), near human settlements, in which a population of the Okinawa rail had newly inhabited for feeding, and a forest area (FA), their natural habitat. Among 16 *E. coli*‐positive faecal samples collected around LA, 11/16 (69%) showed antimicrobial resistance and five multiple drug resistance patterns were identified. However, among 15 *E. coli*‐positive faecal samples from FA, 3/15 (20%) showed antimicrobial resistance, and three multiple drug resistance patterns were identified. These results indicate that the endangered Okinawa rail may also play an important role as a potential vector for the spread of AMR in the natural environment. To maintain ecological health, it is imperative that in situ/ex situ conservation projects that include translocation plans for endangered species are aware of these data.

## Abstract 2

We identified antimicrobial‐resistant bacteria in Okinawa rails living near a livestock farm and in forest areas in Okinawa Main Island, suggesting that Okinawa rails play a role in spreading antimicrobial resistance between areas of human habitation and the natural environment.

## Graphical abstract

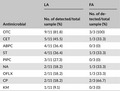

## 1 INTRODUCTION

Antimicrobial resistance (AMR) is a growing social problem for public, animal and environmental health worldwide (World Health Organization, 2014). The use of antimicrobial drugs in livestock farms and in the rearing of domestic animals is suspected to play an important role in spreading AMR (Nhung et al., 2015). Previous studies have shown that AMR in wildlife tends to be more prevalent closer to human settlements where wild animals could have contact with livestock or companion animals in which antibacterial drugs are used as clinical therapies or growth stimulants (Kozak, Boerlin, Janecko, Reid‐Smith, & Jardine, 2009; Yagasaki, 1986). Direct and/or indirect contact among farm or pet animals and wildlife could be involved in the transmission of AMR, facilitating the dissemination of AMR into natural environments. Many attempts are being made to understand how AMR is spread. Antimicrobial‐resistant strains of *Escherichia coli* have been used as an indicator of the routes of AMR spread (Luo et al., 2011; Van den Bogaard & Stobberingh, 2000).

**Figure 1:**
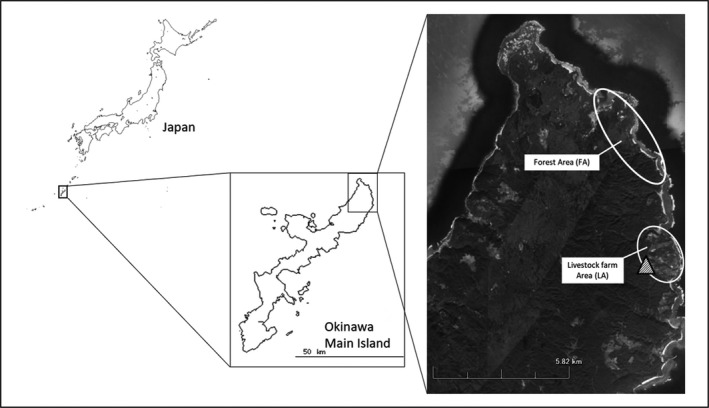
Sampling Area; Livestock farm area (LA) and Forest area (FA) locations in the Kunigami village in Okinawa prefecture. The triangle symbol indicated the livestock farm, and fecal sample were collected on the road within each white circle

The Okinawa rail (*Gallirallus okinawae*) is a flightless bird in the family Rallidae and the species inhabit the evergreen laurel forest zone, called “Yambaru,” located in the northern part of Okinawa Main Island, Japan (Figure 1). Wild populations of the Okinawa rail are declining because of habitat loss and predation by invasive alien species such as the small Indian mongoose (*Herpestes auropunctatus*), feral cats (*Felis catus*) and feral dogs (*Canis lupus*) (Arcilla, Choi, Ozaki, & Lepczyk, 2015; Harato & Ozaki, 1993; Yamada & Sugimura, 2004). The Okinawa rail is currently listed as an endangered species in The International Union for Conservation of Nature Red List (IUCN, 2017) and highly protected under Act on Conservation of Endangered Species of Wild Fauna and Flora and Programs for the Rehabilitation of Natural Habitats and Maintenance of Viable Populations of Japan. Recently, it has been observed that the Okinawa rail population is slightly increasing around some livestock farms, where they possibly have easy access to food sources, including earthworms and insects (Ogura, Iijima, Ozaki, Nagamine, & Kuwana, 2009). Therefore, it is assumed that the Okinawa rail is exposed to AMR through food and through water contaminated with the faeces of livestock animals.

The aim of this study was to understand whether the Okinawa rail, an endangered species, plays an important role in carrying and spreading AMR, acting as a vehicle between human habitation and the natural environment in Okinawa Main Island.

## 2 MATERIALS AND METHODS

Fifty‐nine avian faecal samples which were suspected of wild Okinawa rails were collected between August 2012 and May 2014. The survey was carried out in Kunigami village, Okinawa Main Island, Japan located at 26°45′N and 128°17′E (Figure 1). Freshly excreted faeces on the asphalt road were collected using a sterilized swab or spoon in the early morning. After sampling, the surface of the road at the collection site was washed for the next sampling. Thirty faecal samples (18 samples collected in 2012, 10 samples collected in 2013 and two samples collected in 2014) were collected from around livestock farm areas (abbreviated LA), including human settlements, where Okinawa rails were frequently observed and 29 faecal samples were collected from a forest area (abbreviated FA) in 2014, located about 3–7 km from the LA sites (Figure 1). It is said that the territory size of Okinawa rails is about 1 km, but it is unpublished (private message from observer). Samples were stored at 4°C until laboratory examination.

To identify whether faecal samples were derived from Okinawa rails, we first screened for its characteristic odour in the sampling area. Secondly, molecular methods were employed to confirm that the faecal samples were from the desired species. The white‐breasted waterhen (*Amaurornis phoenicurus*), in the family Rallidae, inhabits the same area as the Okinawa rail and faeces from this species were used for discrimination. White‐breasted waterhen faecal samples were collected from captive individuals at Neo Park Okinawa, Okinawa, Japan, on November 4, 2013. For polymerase chain reaction (PCR) amplification, a primer set targeting a conserved region of the Okinawa rail mitochondrial ATP6 gene was designed using available sequence data (GenBank accession no. AP010821). The primers used were as follows: yanbaru kuina *F* (5′‐ATGGGCCCTAACACTCTCCT‐3′; nucleotides 12,945 to 12,964) and yanbaru kuina R (5′‐GGAGACTGCGGGTATGATGG‐3′; nucleotides 13,311 to 13,292). PCR products were purified using the QIAquick PCR Purification Kit^®^ (QIAGEN, USA) and direct‐sequenced using an ABI 3130xl sequencing system (Applied Biosystems, USA). The described primer set was also used for sequencing.

Okinawa rail faecal samples, identified using PCR, were cultured on MacConkey agar (Nissui Pharmaceutical Co., Ltd., Tokyo, Japan) and screened for *E.* *coli* using Triple Sugar Iron and Motility‐Indole‐Lysine media (Nissui Pharmaceutical Co., Ltd., Tokyo, Japan), followed by identification using the ID‐Test/EB20 (Nissui Pharmaceutical Co., Ltd., Tokyo, Japan). The drug susceptibility of the isolates was determined according to the Clinical and Laboratory Standards Institute methods (Clinical and Laboratory Standards Institute, ). The Kirby–Bauer disk diffusion method was used to determine the antimicrobial agent sensitivity profiles of *E.* *coli* isolates for the following 17 antimicrobial agents: Ampicillin (ABPC), Piperacillin (PIPC), Cefozopran (CZOP), Kanamycin (KM), Gentamicin (GM), Oxytetracycline (OTC), Ofloxacin (OFLX), Chloramphenicol (CP), Nalidixic acid (NA), Fosfomycin (FOM), Sulfamethoxazole‐trimethoprim (ST), Streptomycin (SM), Ceftazidime (CAZ), Ceftriaxone (CTRX), Cefotaxime (CTX), Cephalothin (CET) and Cefuroxime (CXM) (Clinical and Laboratory Standards Institute, ). These antimicrobial drugs were chosen based on their clinical and agricultural uses for domestic animals and as feed additives. The diameters (in millimetres) of the clear zones of growth inhibition around the antimicrobial agent disks were measured using precision calipers. To categorize isolates as resistant or non‐resistant to each antimicrobial agent, we used the standard method (Clinical and Laboratory Standards Institute, ). The *E. coli* strain ATCC 25,922 (American Type Culture Collection) was used for quality control.

The minimum inhibitory concentration (MIC) was determined using the ETEST^®^ (SYSMEX bioMérieux Co., Ltd.), according to the manufacturer's recommendations. The inoculum was matched to a McFarland standard of 0.5, and, using a sterilization swab, a lawn of bacteria was created on Mueller‐Hinton agar plates. Two ETEST^®^ strips were placed onto each Mueller‐Hinton agar plate. The plates were incubated for 18 hr at 35°C. The MIC was interpreted as the value at which the inhibition zone intersected the scale on the ETEST^®^ strip.

Chi‐squared testing was used to compare the prevalence of antimicrobial‐resistant bacteria from faecal samples obtained from the LA and FA samples. Antimicrobial resistance was expressed as odds ratios with 95% confidence intervals.

## 3 RESULTS AND DISCUSSION

For species identification, the Okinawa rail mitochondrial ATP6 gene was successfully amplified using PCR in 81% (48/59) of faecal samples. Of these 48 samples, 22 samples were collected from LA and 26 samples collected from FA.

*E. coli* were isolated from 31 of 48 (65%) samples, and 14 of 31 (45%) samples were antimicrobial resistant. Among the 22 faecal samples collected around LA, *E.* *coli* was isolated from 16 (73%) and 11 (69%) of 16 samples showed antimicrobial resistance. *E.* *coli* was isolated from 15 (58%) of the 26 faecal samples collected around FA and three (20%) of the 15 *E.* *coli*‐positive samples showed antimicrobial resistance (Table 1). The prevalence of AMR *E.* *coli* in faecal samples from wild Okinawa rails from LA (69%) was significantly higher than those from FA (20%) (*p* = .006). The MICs of antimicrobials (in μg/ml) are shown in Table 2. Furthermore, five different multiple drug resistance (MDR) patterns were found in LA samples and three different MDR patterns were found in FA samples (Table 3).

**Table 1:** Proportion of Okinawa Rail fecal samples demonstrating resistance to each antimicrobial. No resistance to CZOP, GM, FOM, SM, CAZ, CTRX, CTX and CXM was detected in *Escherichia coli* isolates from Okinawa rail faecal samples in both areas

Antimicrobial	LA	FA
	No. of detected/total sample (%)	No. of detected/total sample (%)
OTC	9/11 (81.8)	3/3 (100)
CET	5/11 (45.5)	1/3 (33.3)
ABPC	4/11 (36.4)	0/3 (0)
ST	4/11 (36.4)	1/3 (33.3)
PIPC	3/11 (27.3)	0/3 (0)
NA	2/11 (18.2)	1/3 (33.3)
OFLX	2/11 (18.2)	1/3 (33.3)
CP	2/11 (18.2)	2/3 (66.7)
KM	1/11 (9.1)	0/3 (0)

**Table 2:** MICs of AMR *E. coli* from Okinawa Rail faecal samples in LA and FA

Antimicrobial	Sampling area	MIC (µg/ml)							
		1	8	16	32	64	96	128	≧256
OTC	FA	1				1		1	
	LA							2	7
CET	FA				1				
	LA		1	1	1		1		1
ABPC	FA								
	LA								4
ST	FA				1				
	LA				4				
PIPC	FA								
	LA				2				1
NA	FA								1
	LA								2
OFLX	FA				1				
	LA				2				
CP	FA								2
	LA								2
KM	FA								
	LA								1

**Table 3:** MDR patterns of AMR *E.* *coli* isolated from faecal samples of Okinawa rail collected in LA and FA

Sampling area	Resistance pattern	Resistance antimicrobial	No. of detected samples
LA	Single‐drug resistance	OTC	4
		CET	2
	Multi‐drug resistance	OTC‐ST	1
		ABPC‐PIPC‐KM‐OTC‐CET	1
		ABPC‐PIPC‐OTC‐ST‐CET	1
		ABPC‐OTC‐NA‐OFLX‐CP‐ST	1
		ABPC‐PIPC‐OTC‐NA‐OFLX‐CP‐ST‐CET	1
FA	Multi‐drug resistance	OTC‐CP	1
		OTC‐CET	1
		OTC‐NA‐OFLX‐CP‐ST	1

Previous research investigating AMR in Japanese wild birds revealed that antimicrobial‐resistant strains of *E.* *coli* were found in 12.5% of wild green pheasants (*Phasianus versicolor*) and in 15.8% of wild bamboo partridges (*Bambusicola thoracicus*) (Kanai, Hashimoto, & Mitsuhashi, 1981; Nakamura, Yoshimura, & Koeda, 1982). By comparison, the prevalence of AMR in Okinawa rails from LA was much higher, and it is likely that livestock farms could be a source of AMR exposure. Indeed, it is indicated that resistant strains of bacteria which was produced in the livestock body by inadequate use of antibiotics for the treatment of clinical disease, as feed additives for the prevention of disease, or for nutritional purposes (Blanco, Lemus, & Grande, 2009) and/or which was produced by remained antibiotics in their excrements and feed have mainly emerged from livestock farming to the environment. Our results suggest that Okinawa rails are exposed to AMR *E. coli* at livestock farms. Therefore, it is suspected that the movement of Okinawa rails between LA and FA transmitted AMR.

The most frequently detected AMR strain of *E.* *coli* from wild Okinawa rails was OTC‐resistant. OTC is widely used in the rearing of domestic animals to prevent and treat infectious diseases and to promote their growth (Asai, 2010; Horie & Takegami, 2006). OTC‐resistant strains have been detected in hooded cranes (*Grus monacha*) that inhabit cattle farms in the Kagoshima Prefecture, Japan. The greatest OTC MIC value in hooded cranes was 128 μg/ml (Kitadai, Obi, Yamashita, Murase, & Takase, 2012); in Okinawa rails, we detected a higher resistance to OTC (MIC ≥ 256 μg/ml). Oonaka, Furuhata, Kiuchi, Hara, and Fukuyama (2004) compared AMR bacterium MIC values in samples from the environment and of human clinical origin, and found higher MIC values in human clinical isolates. Although the use of ABPC in human therapy is quite rare (Terada, Miyake, & Urase, 2012), 16 t/year of bulk powder has been sold in Japan for use with domestic and pet animals (National Veterinary Assay Laboratory Ministry of Agriculture, Forestry, & Fisheries of Japan, 2012). OTC and ABPC resistance in *E.* *coli* from wild Okinawa rails inhabiting the area around LA indicates that the livestock industry is likely the source origin of AMR for these antimicrobials. Furthermore, CP resistance was detected in rails collected in FA at high rates (67%). CP has been frequently used as a therapeutic agent for domestic animals in livestock farms in Japan because of its broad‐spectrum activity against pathogenic bacteria (Harada, 2009). However, this drug is toxic to humans, causing aplastic anaemia, and the Japanese government has permanently banned the use of CP for the treatment of disease in food‐producing animals (Gilmore, 1986; Harada, 2009). The presence of CP‐resistant *E.* *coli* isolated from the Okinawa rail suggests that CP‐resistant bacteria may have remained at the sampling areas more than 10 years after the use of the drug had ceased, or that CP may still be used for livestock animals at this location. Moreover, synthetic antimicrobial agents, including NA, OFLX and ST are substances that are created chemically, and are not naturally occurring antibiotics (Ministry of agriculture, forestry, & fisheries of Japan, 2014), and they have remained relatively stable in the environment (Sarmah, Meyer, & Boxall, 2006). There were 2–8 multiple drug resistance patterns, including synthetic antimicrobial agents, in resistant *E.* *coli* isolated from FA and LA.

It is suggested that AMR occurs as a result of interactions between microbial agents, host organisms and the environment (Delport, Harcourt, Beaumont, Webster, & Power, 2015). The indiscriminate use of certain antimicrobials in human and veterinary medicine has become a significant public health concern because it may select for resistant bacterial strains (Hiltunen, Virta, & Laine, 2017). This study indicates that wildlife, even critically endangered species such as the Okinawa rail, may play an important role as host reservoirs and potential vectors for the spread of AMR into the natural environment. However, we assembled samples from different areas in different year and our sample size was small. Therefore, more samples need to be collected and genetic analysis of AMR *E.* *coli* using the PFGE (Pulsed‐Field Gel Electrophoresis) method needs to be performed. Conclusively, it is imperative that attention has to be given to the prevalence of AMR among endangered species in programmes of in situ/ex situ conservation that include reintroduction, in order to maintain public and ecological health.

## CONFLICT OF INTEREST

The authors have no conflict of interests to declare.

## References

[vms3194-bib-0001] Arcilla, N. , Choi, C. Y. , Ozaki, K. , & Lepczyk, C. A. (2015). Invasive species and Pacific island bird conservation: A selective review of recent research featuring case studies of Swinhoe’s storm petrel and the Okinawa and Guam rail. Journal of Ornithology, 156, 199–207. 10.1007/s10336-015-1256-8

[vms3194-bib-0002] Asai, T. (2010). Japanese veterinary antimicrobial resistance monitoring system: JVARM. Journal of Veterinary Epidemiology, 14, 146–147. 10.2743/jve.14.146

[vms3194-bib-0003] Blanco, G. , Lemus, J. A. , & Grande, J. (2009). Microbial pollution in wildlife: Linking agricultural manuring and bacterial antibiotic resistance in red‐billed choughs. Environmental Research, 109, 405–412. 10.1016/j.envres.2009.01.007 19264302

[vms3194-bib-0004] Clinical and Laboratory Standards Institute . (2012 ). Performance Standards for Antimicrobial Disk Susceptibility Tests; Approved Standard—Eleventh Edition. pp. 76.

[vms3194-bib-0005] Delport, T. C. , Harcourt, R. G. , Beaumont, L. J. , Webster, K. N. , & Power, M. L. (2015). Molecular detection of antibiotic‐resistance determinants in *Escherichia coli* isolated from the endangered Australian sea lion (*Neophoca cinerea*). Journal of Wildlife Diseases, 51, 555–563. 10.7589/2014-08-200 25919463

[vms3194-bib-0006] Gilmore, A. (1986). Chloramphenicol and the politics of health. Canadian Medical Association Journal, 134, 423–435. 3942956 PMC1490803

[vms3194-bib-0007] Harada, K. (2009). Studies on association of the veterinary use of antimicrobials with antimicrobial resistance in *Escherichia coli* obtained from food‐producing animals. Annual Report of the National Veterinary Assay Laboratory, 45, 1–11.

[vms3194-bib-0008] Harato, T. , & Ozaki, K. (1993). Roosting behavior of the Okinawa rail. Journal of the Yamashina Institute for Ornithology, 25, 40–53. 10.3312/jyio1952.25.40

[vms3194-bib-0010] Hiltunen, T. , Virta, M. , & Laine, A. L. (2017). Antibiotic resistance in the wild: An eco‐evolutionary perspective. Philosophical Transactions of the Royal Society B, 372, 20160039. 10.1098/rstb.2016.0039 PMC5182435 27920384

[vms3194-bib-0011] Horie, M. , & Takegami, H. (2006). Legal restriction on veterinary drug residues and analysis of residual veterinary drug in food by LC/MS. Journal of the Mass Spectrometry Society of Japan, 54, 91–96. 10.5702/massspec.54.91

[vms3194-bib-0012] IUCN (2017). The 2017 IUCN red list of threatened species. Retrieved from www.iucnredlist.org. Accessed 3rd October 2018.

[vms3194-bib-0013] Kanai, H. , Hashimoto, H. , & Mitsuhashi, S. (1981). Drug‐resistance and conjugative R plasmids in *Escherichia coli* strains isolated from wild birds (Japanese tree sparrows, Green pheasants and Bamboo partridges). Japanese Poultry Science, 18, 234–239. 10.2141/jpsa.18.234

[vms3194-bib-0014] Kitadai, N. , Obi, T. , Yamashita, S. , Murase, T. , & Takase, K. (2012). Antimicrobial susceptibility of *Escherichia coli* isolated from feces of wild cranes migrating to Kagoshima, Japan. Journal of Veterinary Medical Science, 74, 395–397. 10.1292/jvms.11-0220 22075707

[vms3194-bib-0015] Kozak, G. K. , Boerlin, P. , Janecko, N. , Reid‐Smith, R. J. , & Jardine, C. (2009). Antimicrobial resistance in *Escherichia coli* Isolates from swine and wild small mammals in the proximity of swine farms and in natural environments in Ontario, Canada. Applied Environmental Microbiology, 75, 559–566. 10.1128/AEM.01821-08 19047381 PMC2632148

[vms3194-bib-0016] Luo, Y. , Xu, L. , Rysz, M. , Wang, Y. Q. , Zhang, H. , & Alvarez, P. J. J. (2011). Occurrence and transport of tetracycline, sulfonamide, quinolone, and macrolide antibiotics in the Haihe River Basin, China. Environmental Science & Technology, 45, 1827–1833. 10.1021/es104009s 21309601

[vms3194-bib-0017] Ministry of agriculture, forestry and fisheries of Japan (2014). User guidelines of antibiotics for livestock animals. Retrieved from: http://www.maff.go.jp/j/keiei/hoken/saigai_hosyo/s_kokuzi_tuti/pdf/h_261118_siyo_sisin.pdf. Accessed 3rd October 2018. [In Japanese].

[vms3194-bib-0018] Nakamura, M. , Yoshimura, H. , & Koeda, T. (1982). Drug resistance and R plasmids of *Escherichia coli* strains isolated from six species of wild birds. The Japanese Journal of Veterinary Science, 44, 465–471. 10.1292/jvms1939.44.465 6752506

[vms3194-bib-0019] National Veterinary Assay Laboratory Ministry of Agriculture, Forestry and Fisheries of Japan . (2012). Sales amounts and sales volumes (active substance) of antibiotics, synthetic antibacterials, anthelmintics and antiprotozoals. pp. 11.

[vms3194-bib-0020] Nhung, N. T. , Cuong, N. V. , Campbell, J. , Hoa, N. T. , Bryant, J. E. , Truc, V. N. T. , … Carrique‐Mas, J. (2015). High Levels of antimicrobial resistance among *Escherichia coli* isolates from livestock farms and synanthropic rats and shrews in the Mekong delta of Vietnam. Applied and Environmental Microbiology, 81, 812–820. 10.1128/AEM.03366-14 25398864 PMC4292488

[vms3194-bib-0021] Ogura, G. , Iijima, Y. , Ozaki, K. , Nagamine, T. , & Kuwana, T. (2009). Technical development of environmental arrangement for ex situ conservation and rehabilitation program for Okinawa rail. 2006–2008. Report of Ministry of the Environment. (p. 508). [In: Japanese].

[vms3194-bib-0022] Oonaka, K. , Furuhata, K. , Kiuchi, A. , Hara, M. , & Fukuyama, M. (2004). A basic study of *Vibrio vulnificus* infection: Serotyping and drug sensitivity test of environment‐derived strains and human clinical isolates. Kansenshogaku Zasshi, 78, 83–89. 10.11150/kansenshogakuzasshi1970.78.83 15103898

[vms3194-bib-0023] Sarmah, A. K. , Meyer, M. T. , & Boxall, A. B. A. (2006). A global perspective on the use, sales, exposure pathways, occurrence, fate and effects of veterinary antibiotics (VAs). Chemosphere, 65, 725–759. 10.1016/j.chemosphere.2006.03.026 16677683

[vms3194-bib-0024] Terada, S. , Miyake, H. , & Urase, T. (2012). Profile of antibiotic resistance of *Escherichia coli* isolated from different water environments. Journal of Japan Society on Water Environment, 35, 73–80.

[vms3194-bib-0025] Van den Bogaard, A. E. , & Stobberingh, E. E. (2000). Epidemiology of resistance to antibiotics. links between animals and humans. International Journal of Antimicrobial Agents, 14, 327–335. 10794955 10.1016/s0924-8579(00)00145-x

[vms3194-bib-0026] World Health Organization (2014). Antimicrobial resistance: Global report on surveillance 2014. Overview. Retrieved from: http://www.who.int/drugresistance/documents/surveillancereport/en/. Accessed 3rd October 2018.

[vms3194-bib-0027] Yagasaki, T. (1986). Safety and usage of feed additives. Journal of the Food Hygienic Society of Japan, 27, 451–465. 10.3358/shokueishi.27.451

[vms3194-bib-0028] Yamada, F. , & Sugimura, K. (2004). Negative impact of an invasive small indian mongoose *Herpestes javanicus* on native wildlife species and evaluation of a control project in Amami‐Ohshima and Okinawa Islands, Japan. Global Environmental Research, 8, 117–124.

